# Molecular and Bioinformatic Characterization of the Rice *ROOT UV-B SENSITIVE* Gene Family

**DOI:** 10.1186/s12284-016-0127-0

**Published:** 2016-10-12

**Authors:** Ning Yu, Yaping Liang, Xinxiang Peng, Xuewen Hou

**Affiliations:** 1 Research Center of Plant Stress Biology, College of Life Sciences, South-China Agricultural University, Guangzhou, 510642 China; 2 Key Laboratory of Plant Functional Genomics and Biotechnology, Education Department of Guangdong Province, College of Life Sciences, South China Agricultural University, Guangzhou, 510642 China

**Keywords:** DUF647, Expression profile, *Oryza sativa*, *ROOT UV-B SENSITIVE*, Subcellular localization

## Abstract

**Background:**

*ROOT UV-B SENSITIVE* (*RUS*) genes exist in most eukaryotic organisms, and encode proteins that contain a DUF647 (domain of unknown function 647). Although the *RUS* genes are known to play essential roles in *Arabidopsis* seedling development, their precise functions are not well understood in other plants, including rice.

**Findings:**

In this study, six *OsRUS* genes were cloned from rice root and leaf cDNA libraries. Our analysis showed that the sequence and open reading frame of cloned *OsRUS3* cDNA differs from the predictions reported in the RAP-DB and RGAP databases. Public microarray, MPSS, and EST databases were used to analyze the expression profiles of the six *OsRUS* genes. Expression profiles for all *OsRUS* genes at different rice developmental stages were also analyzed by qRT-PCR. The signal peptide, GPI-anchor, transmembrane domain and subcellular localization of OsRUS proteins were predicted by various bioinformatics tools. Furthermore OsRUS1 was determined to be localized to the chloroplast by a protoplast experiment.

**Conclusions:**

All the characterization of the *OsRUS* family generated from this study will provide a crucial foundation from which to further dissect how *OsRUS* genes function in rice development.

**Electronic supplementary material:**

The online version of this article (doi:10.1186/s12284-016-0127-0) contains supplementary material, which is available to authorized users.

## Findings

### Identification and Cloning of *OsRUS* cDNA

*RUS* genes were first identified by Dr. He’s group in *Arabidopsis* (Tong et al. 2008; Leasure et al. 2009), and it was found that AtRUS1 and AtRUS2 play a role in very-low-fluence UVB response and VB6 homeostasis (Leasure et al. 2011). However, Dr. Estelle’s group discovered that the *weak auxin response* mutant *wxr1* and *wxr3* were caused by mutations in *AtRUS2/WXR1* and *AtRUS1/WXR3*, respectively. Their results suggested a role for these two genes in the regulation of polar auxin transport (Ge et al. 2010; Yu et al. 2013). The inconsistencies between the results of these two research groups have not currently be resolved.

**Fig. 1:**
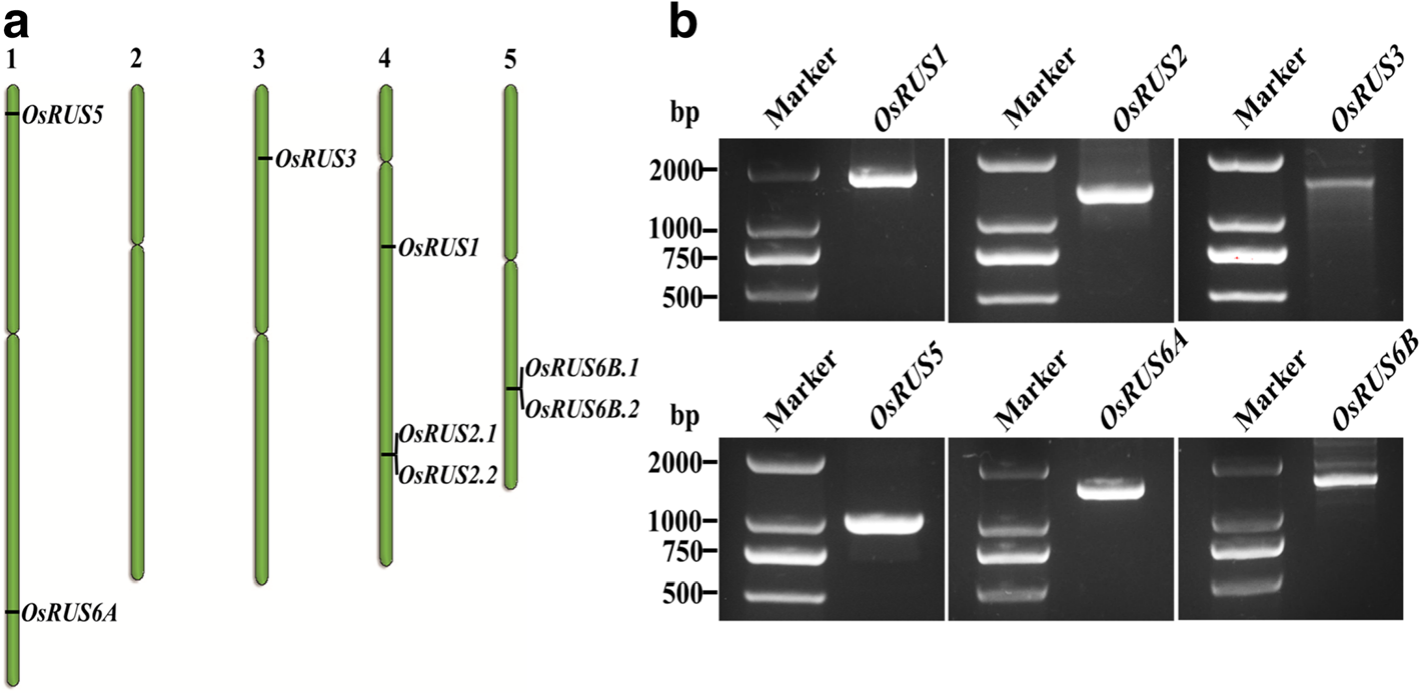
Chromosomal locations of *OsRUS* genes and cloning of the six *OsRUS* cDNAs. **a.** Genomic locations of *OsRUS* genes on rice chromosomes; **b**. Amplification of the six *OsRUS* cDNAs

**Fig. 2:**
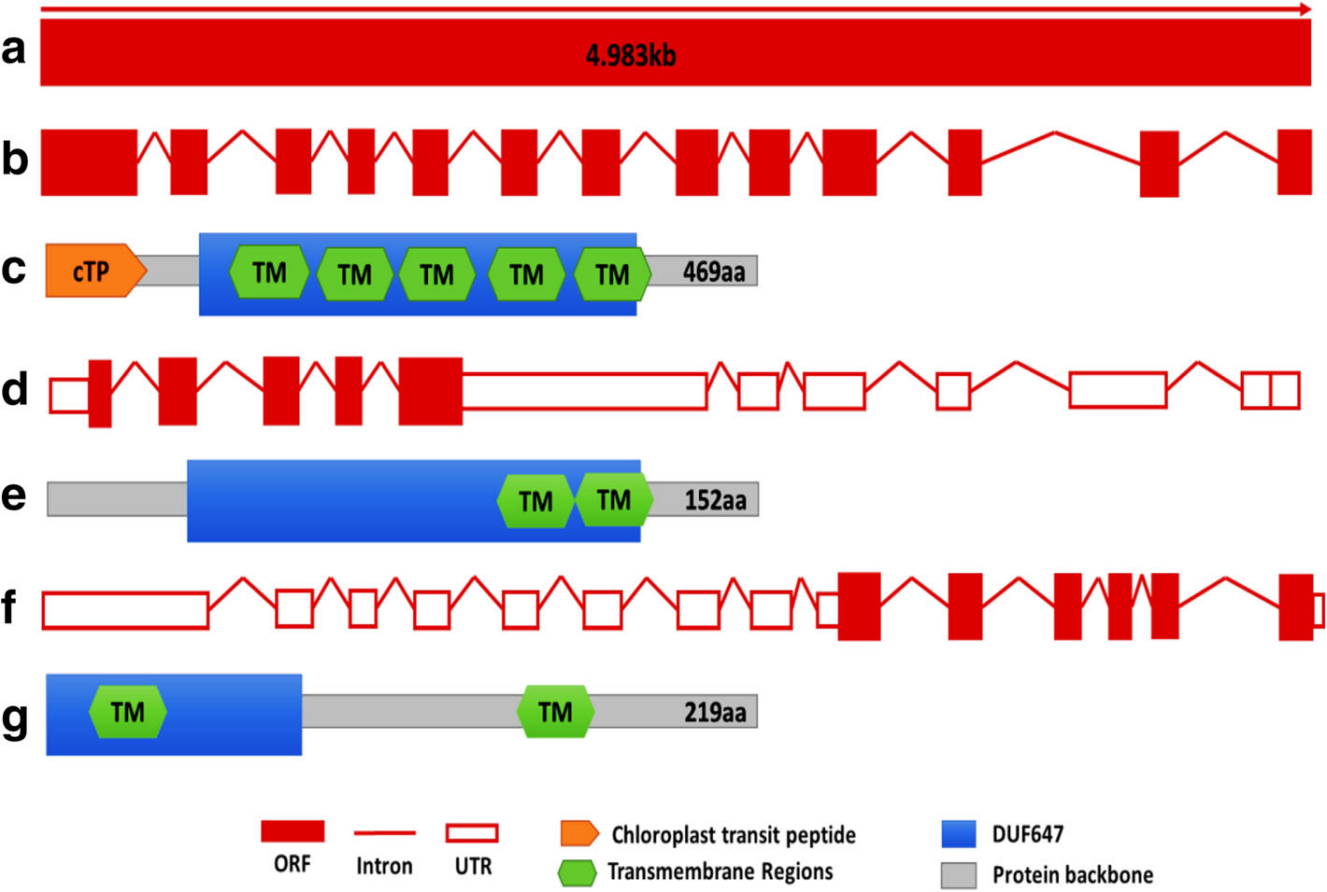
The gene structure comparison of *OsRUS3* generated from cloned sequence, RGAP and RAP-DB databases. **a**. *OsRUS3* genomic DNA; **b**. *OsRUS3* cDNA predicted in the RGAP database; **c**. OsRUS3 protein predicted in the RGAP database; **d**. *OsRUS3* cDNA predicted in the RAP-DB database; **e**. OsRUS3 protein predicted in the RAP-DB database; **f**. Cloned *OsRUS3* cDNA; **g**. OsRUS3 protein translated from cloned *OsRUS3* cDNA. cTP was predicted by ChloroP v1.1; transmembrane domain was predicted by TMpred; DUF647 was predicted by SMART Domain

There are six *AtRUS* genes in the *Arabidopsis* genome, and they all contain a specific domain DUF647. There are six *OsRUS* genes annotated in the rice genome. *OsRUS6* appears to have duplicated in the rice lineage to *OsRUS6A* and *OsRUS6B*, and there is no apparent ortholog for *AtRUS4* (Leasure et al. 2009). The six *OsRUSs* are distributed on four rice chromosomes: *OsRUS*5 and *OsRUS6A* on chromosome 1; *OsRUS*1 and *OsRUS 2* on chromosome 4; *OsRUS3* on chromosome 3; and *OsRUS6B* on chromosome 5 (Fig. 1a). The cDNA library of rice was reverse-transcripted from total RNAs extracted from young seedlings of Zhonghua 11 (Additional file 1: Materials and methods). The primers for cloning the six *OsRUS* cDNAs were designed to amplify their cDNAs (Additional file 2: Table S1). All six *OsRUS* cDNAs were amplified (Fig. 1b), which means that they are all functional genes. The PCR products of the six *OsRUS* cDNAs were cloned and sequenced. Surprisingly the sequence we obtained for the *OsRUS3* cDNA (Additional file 3: Figure S1) was different from the sequences downloaded from the RGAP and RAP-DB databases (Fig. 2b, d and f). All of the other *OsRUS* cDNA sequences were consistent with both databases. The DUF647 domain and transmembrane domains of OsRUS3 were found in the RGAP database, the RAP-DB database and our cloned OsRUS3 (Fig. 2c, e and g). A 56aa cTP was found in the OsRUS3 from RGAP database, but was neither predicted in the OsRUS3 from RAP-DB database nor found in our cloned OsRUS3 (Fig. 2c, e and g). Whether the three types of *OsRUS3* cDNA represent alternative splicing of *LOC_Os03g11500*, or only our cloned cDNA is real, needs further study.

### Expression Profiles of *OsRUS* Genes During Vegetative and Reproductive Development

The expression profiles of genes are highly important for dissecting the functions of the genes (Fang et al. 2016). Here the expression profiles of the six *OsRUS* genes were data-mined from microarray, EST and MPSS publicly available databases and generated by qRT-PCR approach, respectively.

**Fig. 3:**
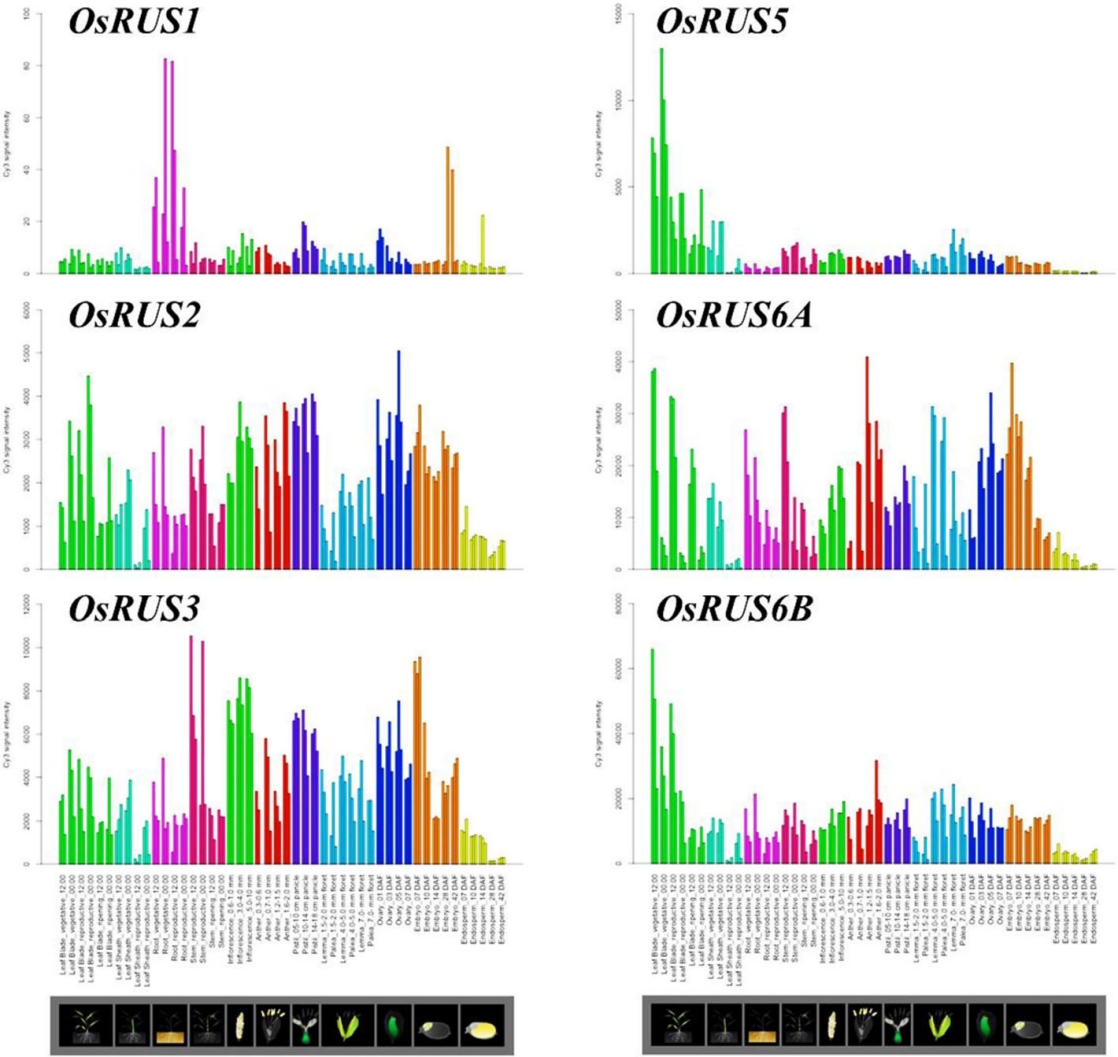
The expression profiles of the six *OsRUS* genes during rice development, data extracted from RiceXPro (http://ricexpro.dna.affrc.go.jp/)

The expression profiles of *OsRUSs* during rice development were extracted from database RiceXPro (http://ricexpro.dna.affrc.go.jp/) (Sato et al. 2011) (Fig. 3). According to this database, the expression level of *OsRUS1* is much higher in roots and late embryos than in other organs. The expression levels of *OsRUS2*, *OsRUS3*, *OsRUS6A* and *OsRUS6B* during rice development are relatively high in all tissues examined, except for in leaf sheath at the reproductive stage and endosperm. The expression level of *OsRUS5* in leaf is much higher than in other organs and stages. These results suggest that *OsRUS2*, *OsRUS3*, *OsRUS6A* and *OsRUS6B* function at similar development stages, while *OsRUS1* and *OsRUS5* function at different stages.

The expression profiles of the *OsRUS* genes were also extracted from the NCBI EST database (http://www.ncbi.nlm.nih.gov/nucest) (Additional file 4: Table S2). The expression of all six *OsRUS* genes can be detected in callus and rice leaf, but the expression level of *OsRUS1*, *OsRUS3* and *OsRUS5* is much lower than that of *OsRUS2*, *OsRUS6A* and *OsRUS6B. OsRUS6B* is not only the sole gene expressed in all of the tissues examined, but also the only *OsRUS* gene expressed in root and SAM, and its expression in SAM is much higher than in other tissues.

According to the information generated from the MPSS database, all six *OsRUS* genes express in callus, all *OsRUS* genes except for *OsRUS2* express in 14d young rice leaves, and all *OsRUS* genes except for *OsRUS1* express in NOS (Ovary and mature stigma) and NIP (90 days - Immature panicle). The expression of *OsRUS1* was only detected in 14d young rice leaves and callus. *OsRUS3* expresses in almost all development stages except for NGS (3 days - Germinating seed). *OsRUS6A* and *OsRUS6B* are highly expressed in all development stages examined. Salt induces the expression of *OsRUS1* in 14d young rice roots and leaves. Cold greatly up-regulates the expression of *OsRUS6A* in 14d young rice leaves. Salt, drought and cold down-regulate the expression of *OsRUS6B* in 14d young rice roots, but highly up-regulate the expression of *OsRUS6B* in 14d young rice leaves (Additional file 5: Table S3).

**Fig. 4:**
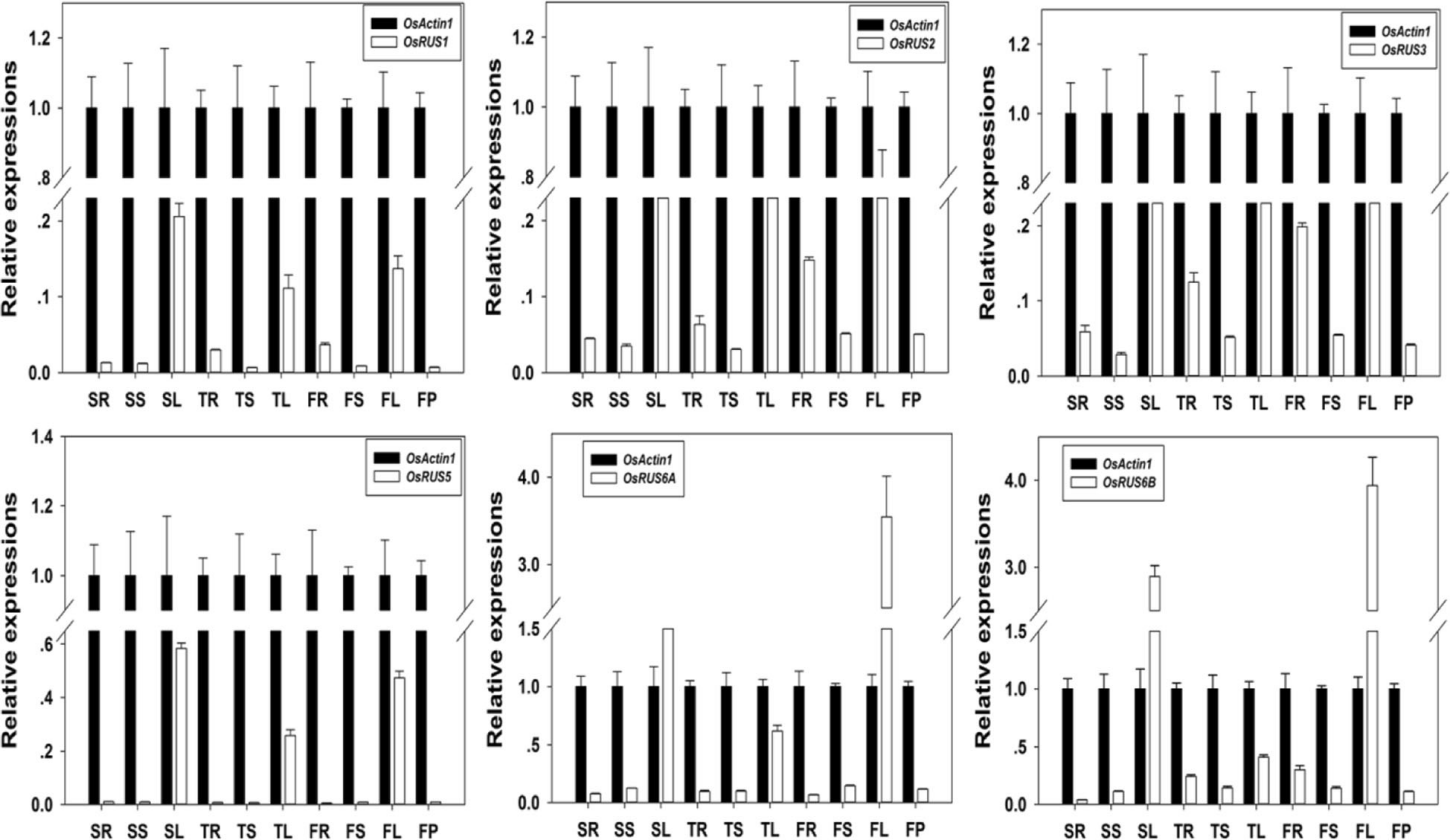
Real-time PCR verification of the expression of *OsRUS* genes in tissues at vegetative and reproductive stages. SR, Root at seeding stage; SL, Leaf at seeding stage; SS, Stem at seeding stage; TR, Root at tillering stage; TL, Leaf at tillering stage; TS, Stem at tillering stage; FR, Root at flowering stage; FL, Leaf at flowering stage; FS, Stem at flowering stage; FP, Panicle at flowering stage

In this paper, qRT-PCR approach was used to verify the expression profiles of the six *OsRUSs* at different rice development stages (Additional file 1: Materials and methods). By using the primers designed for qRT-PCR of six *OsRUSs* (Additional file 6: Table S4), the expression profiles of six *OsRUSs* at different development stages were generated by qRT-PCR (Fig. 4). From the qRT-PCR results, we observed that the six *OsRUS* genes were expressed in all tissues and stages examined. The expression levels of the six *OsRUS* genes in leaves were higher than in other tissues at all stages. Generally speaking, the expression levels of the six *OsRUS* genes were lower than the house-keeping gene *OsACTIN1*, except for *OsRUS6A* and *OsRUS6B* at seedling and flowering stages.

When the expression profiles of OsRUS genes from above three databases and our qRT-PCR experiment were analyzed together, it was found that some results were consistent, while some were not. For example, all six *OsRUS* genes were found to be expressed in all tissues examined in the RiceXPro database and our qRT-PCR experiments. However, only *OsRUS6A* and *OsRUS6B* were found to be expressed in all tissues in the MPSS database, and only *OsRUS6B* was found to be expressed in all tissues in the EST database. The expression level of *OsRUS1* was relatively low in the three databases and the qRT-PCR results. *OsRUS1* expression was only detected in the MPSS database in NYL (14 days Young leaves) and NCA (35 days Callus), and in the EST database it was detected only in callus, leaf, panicle and stem. In the EST database only expression of *OsRUS6B* was detected in roots, while in the MPSS database *OsRUS2*, *OsRUS3*, *OsRUS6A* and *OsRUS6B* were detected in roots. The reasons for this inconsistency are typically complicated, and may be due to cultivar, environment, tissue stage and/or method sensitivity (Ma et al. 2011).

### Subcellular Localization of OsRUS Proteins

The post-translational modifications of a protein are highly important for its function (Guerra et al. 2015). Here the signal peptides (SPs) and GPI-anchor modification signals of the six OsRUSs were predicted by SignalP 4.0 (http://www.cbs.dtu.dk/services/SignalP/) and BigPI (http://mendel.imp.ac.at/gpi/plant_server.html), respectively. None of the OsRUSs was found to have an N-terminal secretion signal (SPs) or a GPI-anchor, indicating that these proteins neither target to the endoplasmic reticulum nor localize to the plasma membrane.

Transmembrane proteins often play important roles in signal transduction or metabolite transport across membranes. Transmembrane domains of OsRUS proteins were predicted using web-based transmembrane domain prediction programs (Additional file 7: Table S5). OsRUS1, OsRUS2, OsRUS3 and OsRUS5 have at least one transmembrane domain predicted by TopPred, TMpred, TMHMM, HMMTOP and SACS HMMTOP tools. OsRUS6A and OsRUS6B have one or three transmembrane domains predicted by TopPred, TMpred, HMMTOP and SACS HMMTOP, but no transmembrane domain predicted by TMHMM. According to the above predictions, OsRUS proteins are likely to be transmembrane proteins.

Determining the subcellular localization of a protein is important for understanding its function. There are many reliable bioinformatics tools available to predict protein subcellular localization. Here the subcellular localizations of OsRUSs were predicted by TargetP, Plant-mPloc, Yloc, ESLpred2, TargetLoc and MultiLoc2 (Table 1), respectively. OsRUS1 and OsRUS5 were predicted to localize to the chloroplast by all six programs used. Although the subcellular localizations of the other OsRUS proteins predicted by the above six programs were not consistent, the chloroplast was the primary predicted subcellular localization: OsRUS2.1 (2/6); OsRUS2.2(4/6); OsRUS3(2/6); OsRUS6A(4/6); OsRUS6B.1(3/6); and OsRUS6B.2 (3/6). The mitochondrion was the second predicted localization for some OsRUS proteins: OsRUS3(2/6): OsRUS6B.1(3/6); and OsRUS6B.2 (3/6).

**Table 1:** Subcellular localizations of OsRUSs predicted by bioinformatics tools

	TargetP	Plant-mPLoc	Yloc	ESLpred2	TargetLoc	MultiLoc2
OsRUS1	Chloroplast	Chloroplast	Chloroplast	Chloroplast	Chloroplast	Chloroplast
OsRUS2.1	Other	Chloroplast	Cytoplasm	Chloroplast	Other	Cytoplasm
OsRUS2.2	Other	Chloroplast	Chloroplast	Chloroplast	Chloroplast	Cytoplasm
OsRUS3	Mitochondrion	Cell membrane	Chloroplast	Chloroplast	Mitochondrion	Secretary pathway
OsRUS5	Chloroplast	Chloroplast	Chloroplast	Chloroplast	Chloroplast	Chloroplast
OsRUS6A	Other	Chloroplast	Chloroplast	Chloroplast	Other	Chloroplast
OsRUS6B.1	Mitochondrion	Chloroplast	Chloroplast	Chloroplast	Mitochondrion	Mitochondrion
OsRUS6B.2	Mitochondrion	Chloroplast	Chloroplast	Chloroplast	Mitochondrion	Mitochondrion

Based on the subcellular localization, non-GPI-anchor modification, and transmembrane predictions, we postulated that OsRUS proteins highly possible localize to the chloroplast membrane.

**Fig. 5:**
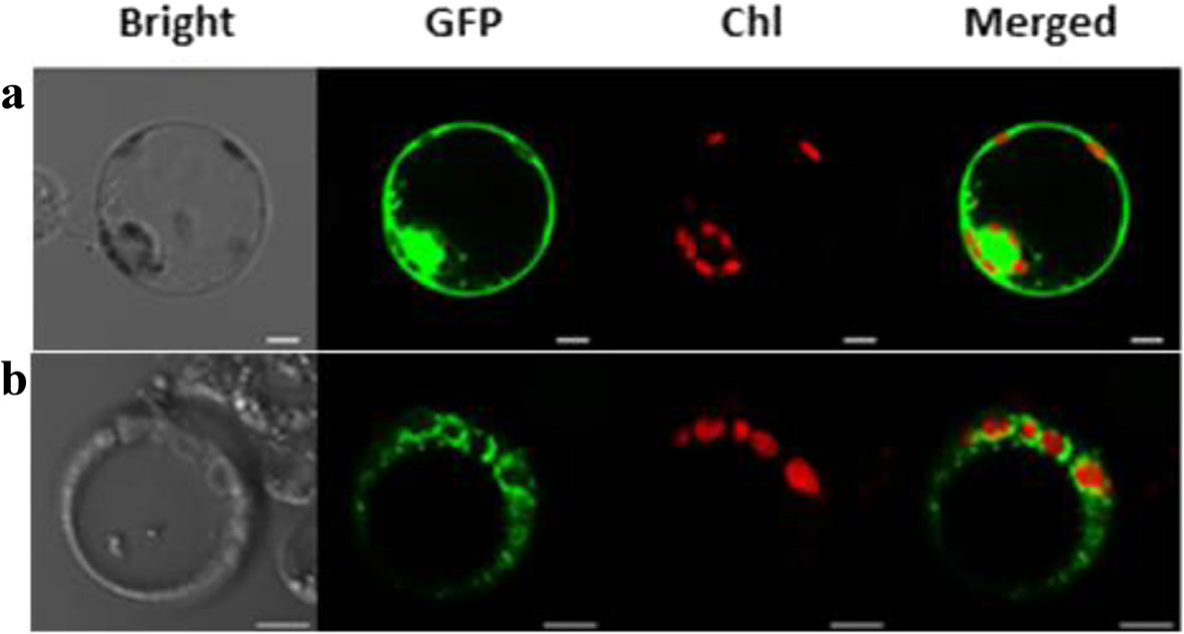
Subcellular localization of OsRUS1 in rice sheath protoplasts. **a**, GFP control. **b**, OsRUS1(1-160aa)::GFP. Individual and merged images of GFP and chlorophyll autofluorescence (Chl), and brightfield (*Bright*) images of protoplasts are shown. Scale bars = 5 μm

In order to evaluate the above subcellular predictions for OsRUS proteins, a protoplast transient-expression approach was used to detect the subcellular localization of OsRUS1 (Additional file 1: Materials and methods). OsRUS1 was predicted to contain a 35aa cTP and be localized to the chloroplast. There is enough information present in the cTP for chloroplast protein sorting (Lee et al. 2008). A transient expression vector of *OsRUS1(1-160aa)::GFP* was constructed and transformed into rice leaf sheath protoplasts. OsRUS1(1-160aa)::GFP was clearly observed to be localized to the chloroplast membrane (Fig. 5b). To our best knowledge this is the first time that the localization of a RUS protein has been experimentally confirmed to be localized to the chloroplast membrane (Tong et al. 2008; Leasure et al. 2009; Ge et al. 2010; Yu et al. 2013).

## Conclusions

There are six *OsRUS* genes in the rice genome, distributed on four chromosomes. The cDNA sequences of five *OsRUS* genes are the same as the predictions of the RGAP and RAP-DB databases, while the cDNA sequence of *OsRUS3* is not. Whether or not this new *OsRUS3* cDNA represents a newly-identified alternative splicing variant has not been resolved. All six OsRUS proteins contain a specific DUF647 domain. The six *OsRUS* genes are expressed in tissues throughout rice development, and they all express more highly in leaves than in other organs. Some *OsRUS* genes have similar expression profiles during rice development. By using available bioinformatics tools, OsRUS proteins are predicted to lack both signal peptides and GPI-anchors, contain transmembrane domains, and be mainly localized to the chloroplast. Combining these predictions together, we postulate that most OsRUS proteins, if not all, localize to the chloroplast membrane. This postulation is supported by the OsRUS1 subcellular localization experiment using a rice protoplast transient-expression approach. All of the work in this paper will support the further dissection of the functions of OsRUS proteins during rice development.

## Additional files

Additional file 1: Materials and methods. (DOCX 27 kb)
Additional file 2: Table S1. Primers for cloning of 6 *OsRUS* cDNAs. (DOCX 16 kb)
Additional file 3: Figure S1. The sequence of cloned *OsRUS3* cDNA. (DOCX 15 kb)
Additional file 4: Table S2. The expression profiles of *OsRUS* genes from NCBI EST database. (DOCX 14 kb)
Additional file 5: Table S3. The expression profiles of *OsRUS* genes from the MPSS database. (DOCX 15 kb)
Additional file 6: Table S4. Primers for qRT-PCR of *OsRUS* genes. (DOCX 15 kb)
Additional file 7: Table S5. Transmembrane domains of OsRUSs predicted by bioinformatics tools. (DOCX 14 kb)

## Abbreviations

cTP: Chloroplast transient peptide
DUF647: Domain of unknown function 647
EST: Expressed sequence tag
GFP: Green fluorescent protein
GPI-anchor: Glycosylphosphatidylinositol anchor
MPSS: Massively parallel signature sequencing
NCBI: The National Center for Biotechnology Information
RAP-DB: The rice annotation project database
RGAP: Rice genome annotation project
*RUS*: *ROOT UV-B SENSITIVE*
SAM: Shoot apical meristem
SPs: Signal peptides
TPM: Transcripts per million
UVB: Ultraviolet-B
*wxr*: *weak auxin response*
