# Supplementary material for: Molecular and Bioinformatic Characterization of the Rice ROOT UV-B SENSITIVE Gene Family
Source: Rice (N Y). 2016 Oct 12;9:55. doi: 10.1186/s12284-016-0127-0 (PMC5059228; doi:10.1186/s12284-016-0127-0)
Supplement: Additional file 1: — Materials and methods. (DOCX 27 kb) [file 12284_2016_127_MOESM1_ESM.docx]

**Materials and Methods**

**Identification of *OsRUS* and bioinformatics analysis**

The genomic DNA, cDNA and amino acid sequences of the six *OsRUS* genes were downloaded from RGAP (Rice Genome Annotation Project, <http://rice.plantbiology.msu.edu/>. Accessed 8 April 2012.) and RAP-DB (The Rice Annotation Project Database, <http://rapdb.dna.affrc.go.jp/>. Accessed 8 April 2012.).

All OsRUS proteins were subjected to SignalP 4.1 (<http://www.cbs.dtu.dk/services/SignalP/>. Accessed 15 March 2016.)(Petersen et al. 2011), and Big-PI Plant Predictor (<http://mendel.imp.ac.at/gpi/plant_server.html>. Accessed 15 January 2016.) (Eisenhaber et al. 2003) to check for the presence of N-terminal signal peptides and GPI-anchor modification signals.

Subcellular localizations of the six OsRUS proteins were predicted by TargetP v1.1 (<http://www.cbs.dtu.dk/services/TargetP/>. Accessed 17 Feb 2016.) (Emanuelsson et al. 2007), Plant-mPLoc (<http://www.csbio.sjtu.edu.cn/bioinf/plant-multi/>. Accessed 17 Feb 2016.) (Chou and Shen 2010), Yloc (<http://abi.inf.uni-tuebingen.de/Services/YLoc/webloc.cgi>. Accessed 17 Feb 2016.) (Briesemeister et al. 2010), [ESLpred2](http://www.imtech.res.in/raghava/eslpred2/) (<http://www.imtech.res.in/raghava/eslpred2/submit.html>. Accessed 17 Feb 2016.)(Garg and Raghava 2008), TargetLoc (<http://abi.inf.uni-tuebingen.de/Services/MultiLoc>. Accessed 17 Feb 2016.) (Hoeglund et al. 2006), and MultiLoc2 (<http://abi.inf.uni-tuebingen.de/Services/MultiLoc2>. Accessed 17 Feb 2016.) (Blum et al. 2009).

Transmembrane domains of the six OsRUS proteins were predicted by TopPred (<http://mobyle.pasteur.fr/cgi-bin/portal.py?#forms::toppred>. Accessed 23 Feb 2016.) (Claros and von Heijne 1994), TMpred (<http://www.ch.embnet.org/software/TMPRED_form.html>. Accessed 23 Feb 2016.) (Hofmann [and Stoffel](../../../../Desktop/Downloads/and%20Stoffel,) 1993), TMHMM (<http://www.cbs.dtu.dk/services/TMHMM/>. Accessed 23 Feb 2016.) (Krogh et al. 2001), HMMTOP (<http://www.enzim.hu/hmmtop/>. Accessed 23 Feb 2016.) (Tusnády and Simon 2001), and SACS HMMTOP (<http://www.sacs.ucsf.edu/cgi-bin/hmmtop.py>. Accessed 23 Feb 2016.) (Tusnády and Simon 2001).

**Cloning of 6 *OsRUS* gene cDNAs**

Total RNA from 14 days old Zhonghua-11 rice seedlings was extracted by using TRIzol reagent (Invitrogen, CA, USA). The RNA samples were incubated at 37°Cfor 30 min with DNase I (RNase-free) (Takara, Dalian, China) to remove DNA contamination prior to RNA reverse transcription. Then, 1μl EDTA (50 mM) was added to the mixture and incubated at 65°C for 10 min to inactivate DNase I. The cDNA was synthesized from 1 μg total RNA by using Reverse Transcriptase M-MLV(RNase H^-^) (Takara, Dalian, China). The six *OsRUS* gene cDNAs were amplified by using KOD Plus neo polymerase (Toyobo, Shanghai, China) and the primers shown in Table S1, respectively. The amplified PCR products were cloned into pMD 18-T simple vector (Takara, Dalian, China) after A-tailing. The positive clones were sent to Taihe Biotechnology Co., LTD (Guangzhou, China) for sequencing. The cloned sequences were compared with sequences downloaded from RGAP and RAP-DB.

**Chromosomal localization of *OsRUS* genes**

The chromosomal locations of *OsRUS* genes were mapped on the physical maps of rice chromosomes using the Chromosome Map Tool at the Oryzabase website (<http://www.shigen.nig.ac.jp/rice/oryzabase/top/top.jsp>. Accessed 16 Jan 2016.).

**Digital expression analysis of *OsRUS* genes**

The expression profiles of the six *OsRUS* genes were extracted from the microarray database RiceXPro (<http://ricexpro.dna.affrc.go.jp/>. Accessed 22 Feb 2016.) (Sato et al. 2011).

EST (expressed sequence tag) expression profiles of *OsRUS* genes were obtained from UniGene database at NCBI(<http://www.ncbi.nlm.nih.gov/nucest>. Accessed 29 Nov 2015.). Genes were defined as specifically expressed if the EST number from any tissue contributed more than half of the total frequency.

MPSS tags of *OsRUS* genes were obtained from the MPSS project (<http://mpss.udel.edu>. Accessed 30 Nov 2015.) (Nakano et al. 2005) and mapped to TIGR gene models. If the signature uniquely identified an individual gene and showed perfect match, then it was considered significant. For a given gene in a given library, the normalized abundance (TPM, tags per million) of these signatures indicated a quantitative expression of that gene. MPSS expression data for 17- and 20-base signatures demonstrating different tissues and treatment were used for *OsRUS* genes expression analysis.

**Quantitative real-time RT-PCR analysis of *OsRUS* genes**

The method of Hou et al. (2005) was adopted. Total RNA from tissues of different growth stages of ZhongHua11 under sunlight were extracted using TRIzol solution (Invitrogen, CA, USA). cDNAs were synthesized from 1μg of total RNA using a Reverse Transcriptase M-MLV(RNase H^-^) (Takara, Dalian, China). Quantitative RT-PCR reaction was performed using the Biotool^TM^ 2x SYBR Green QPCR Master Mix (Biotool, Shanghai, China) on the CFX96 real-time system (Bio-Rad, CA, USA) following the manufacturer’s instructions. The rice *ACTIN1* gene was amplified as an internal standard to the expression of *OsRUS* genes. All the primers used to test the expression of *OsRUS* genes are listed in Table S3.

**Construction of transient expression vector pYL322-dl***-****OsRUS1(1-160aa)::eGFP***

The fragment of 499bp including the cTP was cloned by using primer pair (*OsRUS1* ORF-*Eco*RI-1-F: 5′-AGAATTCATGTCCTCCTCGCAATCTCTCC-3′ and *OsRUS1* ORF-*Bam*HI-498-R: 5′- TGGATCCCCTTCTCCGCATCCTCC-3′) and KOD Plus neo polymerase. After confirmed by sequencing, it was subcloned into pYL322-dl-*eGFP* (Zhu et al. 2014) to form pYL322-dl-*OsRUS1(1-160aa)::eGFP*.

**Subcellular localization of OsRUS1 by protoplast transient expression**

The method of Zhang et al. (2011) was adopted. Briefly, moderate plump seeds of ZhongHua11 were dipped in distilled water for 36 h. These seeds were put on a plate with two humid filter papers, and then the plate was placed in a 28°C dark incubator for 48 h to germinate seeds. The germinated seeds were sowed in slurry soil, and grown under natural conditions for 10-15 days. The sheath of 30-50 rice seedlings were cut into approximately 1-2 mm strips using razors. The strips were transferred into an enzyme solution (1.5% Cellulase, 0.75% Macerozyme, 0.6M mannitol, 10mM MES at pH5.7, 10mM CaCl_2_, 0.1% BSA and 100 mg/ml Ampicillin) at once, and then digested for 4-5 hours at 25°C in the dark with 40 rpm gentle shaking. After the enzymolysis, the same volume of W5 solution (154mM NaCl, 125mM CaCl_2_, 5mM KCl and 2mM MES at pH 5.7) was added into the enzyme solution, and gently shook for an additional 30 minutes to fully release the protoplasts. Protoplasts were filtered through 75 μm nylon mesh into 30ml round bottom tubes, and collected by centrifugation at 250g for 5 minutes. 1 ml W5 solution was added to resuspend the pellets, and then transferred to 2ml tubes, centrifugation at 800 rpm for 5 minutes. The pellets were resuspended in MMG solution (0.4M mannitol, 15mM MgCl_2_ and 4mM MES at pH5.7) at a concentration of 2 x 10^6^ cells mL^-1^, determined by using a hemacytometer. 10-15 μg of plasmid DNA were added in a 2ml tube, and gently mixed with 100 μL of protoplasts. Then, an equal volume of freshly prepared PEG solution (40% PEG 4000, 0.2mM mannitol and 0.1mM CaCl_2_) was added and immediately gently mixed to uniformity. The mixture was incubated at room temperature for 20 minutes in the dark. After that, a 2-fold volume of W5 solution was slowly added and mixed well by gently inverting the tube, followed by centrifugation at 250g for 5 minutes to collect the protoplasts. The pellets were gently resuspended in 1 ml WI solution (0.5mM mannitol, 20mM KCl and 4mM MES at pH 5.7). Finally, the protoplasts were transferred into six well culture plates and cultured in the dark at 28°C for 6-16 hours. All above manipulations were performed at room temperature. The GFP fluorescence of transformed protoplast was detected by LSM780 (ZEISS, Jena, Germany).

**References:**

Blum T, Briesemeister S, Kohlbacher O (2009) [MultiLoc2: integrating phylogeny and Gene Ontology terms improves subcellular protein localization prediction.](http://abi.inf.uni-tuebingen.de/Publications/MultiLoc2) BMC Bioinformatics 10:274

Briesemeister S, Rahnenführer J, Kohlbacher O (2010) YLoc - an interpretable web server for predicting subcellular localization. Nucleic Acids Res 38:W497-W502

Chou KC, Shen HB (2010) Plant-mPLoc: a top-down strategy to augment the power for predicting plant protein subcellular localization. PLoS ONE **5**: e11335

Claros MG and von Heijne G (1994) TopPred II: An improved software for membrane protein structure predictions. CABIOS 10: 685-686

Eisenhaber B, Wildpaner M, Schultz CJ, Borner GHH, Dupree P, Eisenhaber F (2003) Glycosylphosphatidylinositol lipid anchoring of plant proteins. Sensitive prediction from sequence- and genome-wide studies for *Arabidopsis* and rice. Plant Physiol 133:1691-1701

Emanuelsson O, Brunak S, von Heijne G, Nielsen Hk (2007) Locating proteins in the cell using TargetP, SignalP, and related tools. Nat Protoc 2: 953-971

Garg A, Raghava GPS (2008) ESLpred2: Improved method for predicting subcellular localization of eukaryotic proteins. [BMC Bioinformatics 9: 503](http://www.biomedcentral.com/1471-2105/9/503/abstract)

Hoeglund A, Doennes P, Blum T, Adolph HW, Kohlbacher O (2006) [MultiLoc: prediction of protein subcellular localization using N-terminal targeting sequences, sequence motifs, and amino acid composition](http://abi.inf.uni-tuebingen.de/Publications/MultiLocGCB/). Bioinformatics 22: 1158-1165.

Hofmann [K, Stoffel W](http://www.ch.embnet.org/documentation/mfc-35.pdf) (1993) TMbase - A database of membrane spanning proteins segments. Biol Chem Hoppe-Seyler **374**: 166

Hou XW, Tong HY, Selby J, DeWitt J, Peng XX, He ZH (2005) Involvement of a cell wall-associated kinase, WAKL4, in *Arabidopsis* mineral responses. Plant Physiol, 139: 1704-1716

Krogh A, Larsson B, von Heijne G, Sonnhammer ELL (2001) Predicting transmembrane protein topology with a hidden Markov model: Application to complete genomes. J Mol Biol 305: 567-580

Lee DW, Kim JK, Lee S, Choi S, Kim S, Hwang I (2008) *Arabidopsis* nuclear-encoded plastid transit peptides contain multiple sequence subgroups with distinctive chloroplast-targeting sequence motifs. Plant Cell 20: 1603–1622

Nakano M, Nobuta K, Vemaraju K, Tej SS, Skogen JW, Meyers BC (2005) Plant MPSS databases: signature-based transcriptional resources for analyses of mRNA and small RNA. Nucleic Acids Res 34: D731-D735

Petersen TN, Brunak S, von Heijne G, Nielsen H (2011) SignalP 4.0: discriminating signal peptides from transmembrane regions. Nat Methods 8:785-786

Sato Y, Antonio B, Namiki N, Motoyama R, Sugimoto K, Takehisa Hinako, Minami H, Kamatsuki K, Kusaba M, Hirochika H, Nagamura Y (2011) Field transcriptome revealed critical developmental and physiological transitions involved in the expression of growth potential in *japonica* rice. BMC Plant Biol 11:10

Tamura K, Dudley J, Nei M, Kumar S (2007) [MEGA4: Molecular evolutionary genetics analysis (MEGA) software version 4.0](http://apps.webofknowledge.com/full_record.do?product=UA&search_mode=GeneralSearch&qid=6&SID=W2KpYgDcNbhdIfvCSw9&page=1&doc=4). Mol Biol Evol 24: 1596-1599

Tusnády GE, Simon I (2001) The HMMTOP transmembrane topology prediction server. Bioinformatics 17: 849-850

Zhu Q, Yang Z, Zhang Q, Chen L, Liu YG (2014) Robust multi-type plasmid modification based on isothermal in vitro recombination. Gene 548: 39-42
