# Supplementary material for: Molecular and Bioinformatic Characterization of the Rice ROOT UV-B SENSITIVE Gene Family
Source: Rice (N Y). 2016 Oct 12;9:55. doi: 10.1186/s12284-016-0127-0 (PMC5059228; doi:10.1186/s12284-016-0127-0)
Supplement: Additional file 2: Table S1. — Primers for cloning of 6 OsRUS cDNAs. (DOCX 16 kb) [file 12284_2016_127_MOESM2_ESM.docx]

**Table S1. Primers for cloning 6 *OsRUS* cDNAs**

| Primer name | Sequence(5'-3') |
| --- | --- |
| *OsRUS1* cDNA -*Bam*HI-1-F | AGGATCCATGTCCTCCTCGCAATCTCTCC |
| *OsRUS1* cDNA-*Mlu*I-1782-R | GACGCGTTATGAAGGAGCATCTCCTATG |
| *OsRUS2.1* cDNA -P-8-*Bam*HI-F | TTGGATCCTCCCGAATGAACATAC |
| *OsRUS2.1* cDNA -1422-*Hin*dIII-R | ACAAAGCTTAAAGCACTACCGCTGAT |
| *OsRUS3* cDNA -1-*Bam*HI-F | AGGATCCATGCACACTAGTCCGATTGTCAT |
| *OsRUS3* cDNA -1434-*Mlu*I-R | AACGCGTGCTACCTTCGCCTGCTTCCTCCT |
| *OsRUS5* cDNA -P-24-*Spe*I-F | AACTAGTGGCGATAGCACCGCACCGGAG |
| *OsRUS5* cDNA -972-*Mlu*I-R | TACGCGTTACCTTACTGTGCGGAATTTGA |
| *OsRUS6A* cDNA -1-*Spe*I-F | TACTAGTATGGCGCCGACGGTGGG |
| *OsRUS6A* cDNA -1531-*Hin*dIII-R | TAAGCTTGGCCTTGCAGGACACA |
| *OsRUS6B.1* cDNA -P-18-*Bam*HI-F | AGGATCCCAACCCACCGCCGCACCGGAGAT |
| *OsRUS6B.1* cDNA -1579-*Mlu*I-R | GACGCGTATTGGGTCATTGAGGTACTATTCC |

**Note: nucleotides underlined are the introduced restriction enzymes recognition sites.**
