# Supplementary material for: Molecular and Bioinformatic Characterization of the Rice ROOT UV-B SENSITIVE Gene Family
Source: Rice (N Y). 2016 Oct 12;9:55. doi: 10.1186/s12284-016-0127-0 (PMC5059228; doi:10.1186/s12284-016-0127-0)
Supplement: Additional file 3: Figure S1. — The sequence of cloned OsRUS3 cDNA. (DOCX 15 kb) [file 12284_2016_127_MOESM3_ESM.docx]

GGATCCATGCACACTAGTCCGATTGTCATTAGCACACGTGAGTCTGACAAAAAGAAGCAGAAACTCCACCAGTCTCCTCGTCTTGAGCTGAGCAGAAGCCCGCGACGTGCCCTGCACTGCACGCGGAAGCAGAGCTCCGAGCCGTGGCGGCGGCGGGCGCGGGGCGGCGTCCGATCCAGATCCGCGCCGCCGGCGTGGGCGGCCGGCTTACGAGGTGGTGGGCGACTGGTTGGCATCATGGACTCCTCCCGCTTCCGGAGTGCGGCGGCCACGGAGGCGGCGGAGGAGGAGGCGGCGGGGTGGGTGACGGTCGAGGAGTGGGCGGGCTCCTCCGCCGCCGCGCTCTCCCGCACCGCCGTCCTCACCGCCTCCCCCTCCTCCTCCCTCGCCTCCCGCAGGTGAACCCCCGTTCCTCCTCCCTCGGATCCGCTTTGCCCCCGCTCACCCATCTCACCCCGCGACGCGGCGGCGGCGCGCGCAGGTTCGGAAGCCGGTGGGGGCGGGTCGGCGGCCGGCTGCTCGGCGCCTTCGTGCCCGAGGGCTTTCCTGGGAGCGTCACTCCGGATTACGTCCCGTTCCAGATGTGGGATACCTTGCAGGGTCTCTCGACTTACATCCGTGCAATGCTGTCTACTCAAGCTCTTTTAGGCGCTATTGGAGTAGGTGAGAAATCTGCCACGGTTATAGGTGCCACTTTTCAGTGGTTTCTCAGGGATTTGACAGGAATGCTCGGTGGTATACTGTTCACTTTTTATCAGGGATCTAACCTTGATAGCAATGCTAAAATGTGGCGCCTAGTTGCAGACTTTATGAATGACCTTGGGATGTTGATGGATCTCTTAAGCCCTCTGTTTCCTTCATCATTGATTGTTATAATGTGTTTAGGCAGCCTATCTCGATCCTTCACTGGTGTTGCTAGTGGAGCAACTAGAGCAGCGTTAACCCAGCATTTTGCACTTGCCAATAATGCAGCTGATATATCTGCAAAGAAGCATACTCAACTTTTCAGGAGGGCAGTCAGGAGACACTTGCAACAATGTTAGGAATGGGACTGGGAATGCTTCTTGCCCATGTTACCAGAGGGCATGCTTTGGGTGTATGGGTTTCATTCCTTTCTCTAACGATATTCCATATGTATGCAAATTACAAGGCAGTGCAGTCACTTTCACTCACAACACTAAATTATGAGAGAAGTTCCATCCTGCTGCAGTACTTCATGGACAATGGTGAAGTCCTCACACCACAACAGGTTTCCAAGCAAGAGCATATTCTTCCATATTGGTCAAGCTGGCGGAAATTTCTTAGAATTAAACTGCCACATGAGCATGTACATTTAGGTGCTAAAGCCTCAAGGCTTACACACCCTGATTTGCTGCAGATTGCAAAAACAAGGCCCTACTATAGAAATGAGAACTATTTCTTGCTGGATAAGGAAGGCAGTGTTCACATTTTCATCCACAAGCAGGCAGCGGCAACAGATATTTTGATGTCCTTCATACATGGACTCGTGTTAGCACATTTGATGCAGAAGAGCAAATCTGGGCATGCGGAGGCTCGCCAATGGATTGATGAGAAATATAACACTTTTATCTCAAAGTTGCAAGTTGAAGGTTACTCAACAGAGCGACTTCTTTCACACTCGATCGTGTGGAGGGCACATTGGGTTCATGGTCCCTCTGAAGAGAAGCTCGAGTAGGAGGAAGCAGGCGAAGGTAGCACGCGT

**Figure S1. The cloned sequence of *OsRUS3* cDNA**.

Underlined is the introduced *Bam*HI and *Mlu*I recognition site, respectively. Nucleotides in gray are the predicted longest *OsRUS3* open reading frame.
