# Supplementary material for: Molecular and Bioinformatic Characterization of the Rice ROOT UV-B SENSITIVE Gene Family
Source: Rice (N Y). 2016 Oct 12;9:55. doi: 10.1186/s12284-016-0127-0 (PMC5059228; doi:10.1186/s12284-016-0127-0)
Supplement: Additional file 4: Table S2. — The expression profiles of OsRUS genes from NCBI EST database. (DOCX 14 kb) [file 12284_2016_127_MOESM4_ESM.docx]

**Table S2. The expression profiles of *OsRUS* genes from NCBI EST database**

| **Gene** | **Locus** | **C** | **F** | **P** | **L** | **R** | **S** | **St** | **SAM** |
| --- | --- | --- | --- | --- | --- | --- | --- | --- | --- |
| ***OsRUS1*** | Os04g0290800 | 18 | - | 28 | 34 | - | - | 7 | - |
| ***OsRUS2*** | Os04g0517300 | 109 | 36 | 7 | 139 | - | 61 | - | - |
| ***OsRUS3*** | Os03g0213700 | 30 | 183 | 21 | 5 | - | - | 31 | - |
| ***OsRUS5*** | Os01g0141600 | 12 | - | - | 5 | - | - | 7 | - |
| ***OsRUS6A*** | Os01g0886900 | 84 | 65 | 36 | 87 | - | 92 | 31 | - |
| ***OsRUS6B*** | Os05g0419200 | 91 | 146 | 79 | 58 | 73 | 61 | 71 | 436 |
| C, callus; F, flower; P, panicle; L, leaf; R, root; S, seed; St, stem; SAM, shoot apical meristem. “-” represents “not detected”. Units---Transcripts per million (TPM). | | | | | | | | | |
