# Supplementary material for: Molecular and Bioinformatic Characterization of the Rice ROOT UV-B SENSITIVE Gene Family
Source: Rice (N Y). 2016 Oct 12;9:55. doi: 10.1186/s12284-016-0127-0 (PMC5059228; doi:10.1186/s12284-016-0127-0)
Supplement: Additional file 5: Table S3. — The expression profiles of OsRUS genes from the MPSS database. (DOCX 15 kb) [file 12284_2016_127_MOESM5_ESM.docx]

**Table S3. The expression profiles of *OsRUS* genes from the MPSS database**

| **Gene** | **NYR** | **NGD** | **NST** | **NYL** | **NME** | **NPO** | **NOS** | **NIP** | **NGS** | **NCA** | **NSR** | **NSL** | **NDR** | **NDL** | **NCR** | **NCL** |
| --- | --- | --- | --- | --- | --- | --- | --- | --- | --- | --- | --- | --- | --- | --- | --- | --- |
| ***OsRUS1*** | 0 | 0 | 0 | 8 | 0 | 0 | 0 | 0 | 0 | 9 | 88 | 24 | 0 | 0 | 0 | 13 |
| ***OsRUS2*** | 43 | 0 | 0 | 0 | 0 | 118 | 62 | 60 | 0 | 8 | 14 | 64 | 48 | 25 | 0 | 29 |
| ***OsRUS3*** | 16 | 236 | 64 | 84 | 621 | 78 | 62 | 42 | 0 | 60 | 34 | 76 | 43 | 139 | 0 | 47 |
| ***OsRUS5*** | 0 | 2 | 1 | 75 | 0 | 0 | 7 | 26 | 16 | 5 | 5 | 195 | 24 | 219 | 0 | 4 |
| ***OsRUS6a*** | 137 | 247 | 148 | 73 | 320 | 234 | 554 | 402 | 197 | 57 | 186 | 75 | 169 | 79 | 59 | 258 |
| ***OsRUS6b*** | 467 | 28 | 433 | 228 | 312 | 181 | 114 | 244 | 250 | 107 | 164 | 385 | 296 | 339 | 165 | 2095 |

NYR, 14 d young roots; NGD, 10 days - Germinating seedlings grown in dark; NST, 60 days – Stem; NYL, 14 days - Young leaves; NME, 60 days - Crown vegetative meristematic tissue; NPO, Mature Pollen; NOS, Ovary and mature stigma; NIP, 90 days - Immature panicle; NGS, 3 days - Germinating seed; NCA, 35 days – Callus; NSR,14 days - Young roots stressed in 250mM NaCl for 24h; NSL, 14 days - Young leaves stressed in 250mM NaCl for 24h; NDR, 14 days - Young roots stressed in drought for 5 days; NDL, 14 days - Young leaves stressed in drought for 5 days; NCR, 14 days - Young roots stressed in 4°C cold for 24h; NCL, 14 days - Young leaves stressed in 4ºC cold for 24h. Units--Tags per million (TPM)**.**
