# Supplementary material for: Molecular and Bioinformatic Characterization of the Rice ROOT UV-B SENSITIVE Gene Family
Source: Rice (N Y). 2016 Oct 12;9:55. doi: 10.1186/s12284-016-0127-0 (PMC5059228; doi:10.1186/s12284-016-0127-0)
Supplement: Additional file 6: Table S4. — Primers for qRT-PCR of OsRUS genes. (DOCX 15 kb) [file 12284_2016_127_MOESM6_ESM.docx]

**Table S4. Primers for qRT-PCR of *OsRUS* genes**

| Primer name | Sequence(5'-3') |
| --- | --- |
| *OsRUS1* ORF-608-F | ACAGCGTGAGCAGCGACTAC |
| *OsRUS1* ORF-720-R | CAATCCAACCGCATACAACA |
| *OsRUS2* ORF-675-F | CAATGTCATGGGAATAGGTG |
| *OsRUS2* ORF-834-R | TCTTTGCGGATTAAGTGTGT |
| *OsRUS3* ORF-210-F | ACAGGTTTCCAAGCAAGAGC |
| *OsRUS3* ORF-340-R | GCAGCAAATCAGGGTGTGTA |
| *OsRUS5* ORF-581-F | CAAAGAAGTGGCGGATGTATG |
| *OsRUS5* ORF-680-R | GACGCAAGTGGGAGGAAGTA |
| *OsRUS6A* ORF-825-F | CACTGCTAAAGGCGAATC |
| *OsRUS6A* ORF-1021-R | CCACCGCAACAGTAAACC |
| *OsRUS6B* ORF-952-F | TTTGCCTTCTTGTCTTGTGG |
| *OsRUS6B* ORF-1105-R | CCTGTGAGTTTCCTTCCTTCA |
| *OsACTIN1-*QF | CTTCATAGGAATGGAAGCTGCGGGTA |
| *OsACTIN1*-QR | CGACCACCTTGATCTTCATGCTGCTA |
