# Supplementary material for: Molecular and Bioinformatic Characterization of the Rice ROOT UV-B SENSITIVE Gene Family
Source: Rice (N Y). 2016 Oct 12;9:55. doi: 10.1186/s12284-016-0127-0 (PMC5059228; doi:10.1186/s12284-016-0127-0)
Supplement: Additional file 7: Table S5. — Transmembrane domains of OsRUSs predicted by bioinformatics tools. (DOCX 14 kb) [file 12284_2016_127_MOESM7_ESM.docx]

**Table S5. Transmembrane domains of OsRUSs predicted by bioinformatics tools**

|  | **TopPred** | **TMpred** | **TMHMM** | **HMMTOP** | **SACS HMMTOP** |
| --- | --- | --- | --- | --- | --- |
| **OsRUS1** | 4, N terminal inside | 4, N terminal inside | 4, N terminal inside | 3, N terminal inside | 4, N terminal inside |
| **OsRUS2** | 4, N terminal outside | 3, N terminal inside | 1, N terminal inside | 3, N terminal outside | 3, N terminal outside |
| **OsRUS3** | 2, N terminal inside | 2, N terminal inside | 2, N terminal inside | 1, N terminal inside | 1, N terminal inside |
| **OsRUS5** | 3, N terminal inside | 4, N terminal inside | 3, N terminal inside | 2, N terminal inside | 2, N terminal inside |
| **OsRUS6A** | 3, N terminal outside | 3, N terminal inside | None | 1, N terminal inside | 1, N terminal inside |
| **0sRUS6B** | 3, N terminal inside | 3, N terminal outside | None | 1, N terminal inside | 1, N terminal inside |
